# Amorphous Calcium Carbonate Shows Anti-Cancer Properties That are Attributed to Its Buffering Capacity

**DOI:** 10.3390/cancers15153785

**Published:** 2023-07-26

**Authors:** Yehudit Natan, Yigal Dov Blum, Amir Arav, Ylena Poliansky, Sara Neuman, Orit Ecker Cohen, Yossi Ben

**Affiliations:** 1 Amorphical Ltd., 11 HaHarash st., Nes-Ziona 7403118, Israel; 2 A.A. Cash Technology Ltd., 59 Shlomzion Hamalka st., Tel-Aviv 6226618, Israel

**Keywords:** cancer, lung cancer, amorphous calcium carbonate, tumor microenvironment, acidosis

## Abstract

**Simple Summary:**

Lung cancer is by far the leading cause of cancer death, making up almost 25% of all cancer deaths. Each year, more people die of lung cancer than colon, breast, and prostate cancers combined. Developing safe new treatments is of great importance. A main feature of solid tumors is their evolved acidic microenvironment. Although this phenomenon has been known for almost 100 years (also known as aerobic glycolysis or the Warburg effect), it was seldom evaluated as a therapeutic target. A recent understanding of the tumor’s microenvironment acidity has shown its impact on disease aggressiveness, the lack of immune system response, drug and irradiation resistance, and overall poor patient prognosis. Amorphous calcium carbonate (ACC) is a non-crystalline form of calcium carbonate, and it is composed of nanoparticles with known buffering capacities. This work describes in vivo and in vitro results showing the anti-cancerous effects of ACC, which are associated with its buffering activity, suggesting a promising therapeutic potential of ACC as a safe treatment for cancer.

**Abstract:**

Aim: Amorphous calcium carbonate (ACC) is a non-crystalline form of calcium carbonate, and it is composed of aggregated nano-size primary particles. Here, we evaluated its anti-cancer effect postulated relative to its buffering capabilities in lung cancer. Methods: Tumors were evaluated in vivo using the Lewis lung carcinoma (LLC) mouse cell line and A549 human lung cancer carcinoma cell line. LLC and A549 cells were injected subcutaneously into the right hind leg of mice. Treatments (ACC, cisplatin, vehicle, and ACC with cisplatin, all given via daily IP injections) started once tumors reached a measurable size. Treatments were carried out for 14 days in the LLC model and for 22 and 24 days in the xenograft model (two experiments). LLC tumors were resected from ACC at the end of the study, and vehicle groups were evaluated for cathepsin B activity. Differential gene expression was carried out on A549 cells following 8 weeks of in vitro culture in the presence or absence of ACC in a culture medium. Results: The ACC treatment decelerated tumor growth rates in both models. When tumor volumes were compared on the last day of each study, the ACC-treated animal tumor volume was reduced by 44.83% compared to vehicle-treated animals in the LLC model. In the xenograft model, the tumor volume was reduced by 51.6% in ACC-treated animals compared to vehicle-treated animals. A more substantial reduction of 74.75% occurred in the combined treatment of ACC and cisplatin compared to the vehicle (carried out only in the LLC model). Cathepsin B activity was significantly reduced in ACC-treated LLC tumors compared to control tumors. Differential gene expression results showed a shift towards anti-tumorigenic pathways in the ACC-treated A549 cells. Conclusion: This study supports the ACC anti-malignant buffering hypothesis by demonstrating decelerated tumor growth, reduced cathepsin B activity, and altered gene expressions to produce anti-cancerous effects.

## 1. Introduction

Cancer is the second cause of death in the US, and lung cancer is the leading cause of mortality among cancer patients [1]. Lung cancer is mainly divided into two types: small cell lung carcinoma and non-small cell lung carcinoma (NSCLC), which is about 80% of all lung cancer cases [2]. The tumor microenvironment of lung cancer (TME), like other solid tumors, is a complex one with regions of aberrant angiogenesis, acidosis, and hypoxia [3,4].

In the microenvironment of the tumors, inflammation and ischemia are often accompanied by a reduced extracellular pH (acidosis) due to a shift in the cells’ metabolic pathway from oxidative phosphorylation (oxphos) to glycolysis [5,6,7,8]. The acidosis phenomenon is derived from metabolic and genetic changes (either mutations or changes in gene expressions) that cancer cells undergo and can cause a shift towards the short metabolic pathway of generating lactate in the cancer cell’s metabolism, even in the presence of sufficient oxygen supply at the mitochondrial level, resulting in the production of protons (hydrogen cations) and lactate. This phenomenon is known as the Warburg Effect, and its causes and mode of countering have been studied for over 90 years [5,6,7,8,9,10,11,12,13,14]. Protons and lactate separate effluxes via an array of acid transporters such as MCT, NHE, and proton pumps, leading to acidosis in the tumor microenvironment (TME) [9]. This decrease in extracellular pH (pHe) prevents intracellular acidity (pHi) in cancer cells [9]. The acidification of the extracellular peritumoral environment is advantageous to the tumor in a multifaceted manner. Tumor microenvironment acidosis increases tumor proliferation, invasion, angiogenesis, and metastasis [13,14,15,16,17,18,19]. TME acidity increases the ability of cancer cells to escape immune responses [20] and causes immunosuppressive effects with respect to CD8+ T cells, dendritic cells (DCs), monocytes, natural killer NKs, and T cell functions [21,22]. TME acidity also results in chemotherapy resistance [23] and radioresistance [24].

Amorphous calcium carbonate (ACC) is the most unstable polymorph of calcium carbonate (CaCO_3_). It is characterized by its inability to polarize light, in contrast to other crystalline polymorphs of calcium carbonate. It is also characterized by the absence of X-ray diffraction (XRD) patterns, suggesting that no long-range order exists in the structure of the salt, and by the low-intensity peak at 1082 cm^−1^ in Raman spectroscopy [25,26]. ACC primary particles fall within the nanometric range between 40 and 100 nm [25,26]. Preclinical studies with radiolabeled calcium in rats showed that ACC had higher solubility and bioavailability compared to crystalline calcium carbonate (CCC) [26]. In an ovariectomized rat model, ACC showed superiority in preventing bone loss compared to commercial CCC and calcium citrate [27]. Additionally, in a clinical study of postmenopausal women, ACC demonstrated superior bioavailability compared to CCC [28].

Recent studies have suggested the use of ACC [29] and other calcium carbonate polymorphs (mainly vaterite) [30] as drug-delivery agents mainly for anticancer treatments. In these studies, ACC or vaterite were loaded with chemotherapy agents (e.g., doxorubicin, cisplatin), which were released once they reached the tumors’ acidic microenvironment. The use of calcium carbonate polymorphs in these studies overlooked the potential of the ACC to act as a therapeutic agent by itself and focused mainly on the controlled release of the cytotoxic chemotherapy agents. Nevertheless, these studies emphasize the safety of calcium carbonate as a delivery system as well.

Modulating the tumor’s acidic microenvironment has been suggested as a potential target for new treatments [10,12,21]. Experimental models have shown the inhibition of metastasis and increased the tumor’s pHe, either by using bicarbonate [31] or a non-volatile buffer [32,33] such as a compound named L-DOS47, which is in its early clinical trial as an agent for TME pHe modulation [34]. A clinical trial with sodium bicarbonate failed to escalate beyond the second dose level, and this was primarily due to its poor taste and grade 1–2 GI disturbances, leading to poor compliance [35]. A study by Som et al. showed that nanosized vaterite was able to elevate the tumor’s pHe and reduced the tumor’s size when administered via IV injections in a mouse model [36].

Although early diagnosis and new treatments improve lung cancer patient prognosis, there is still a need for new treatments, especially those that will have a high safety profile and significantly fewer or milder adverse effects.

ACC is a unique form of calcium carbonate and is, therefore, generally regarded as safe (GRAS) by regulatory agencies. It is already sold as a food supplement [37]. Furthermore, it was found to be safe when evaluated in several clinical studies as a treatment for cancer (NCT03582280 and NCT03057314 [38,39]) and COVID-19 (NCT04900337 [40]). In these studies, patients were treated with ACC given as a sublingual powder, concomitantly with the inhalation of ACC suspensions. These preliminary clinical trials have demonstrated a high safety profile of the treatment albeit the relatively high daily dosage administration.

The present paper describes the use of ACC as a potential treatment in two animal models for lung cancer: the Lewis lung carcinoma (LLC) and a xenograft model with A549 (a human cell line of NSCLC). The tumors’ volume was evaluated during the treatment duration, and cathepsin B activity levels were measured in the LLC model. Additionally, we evaluated the effect of ACC on the gene expression profile of A549 cultured in vitro.

We hypothesize that ACC antitumor effects can be attributed to the ACC’s improved buffering capacity with respect to the tumor’s microenvironment pHe. It is plausible that the disintegration of the salt and subsequent solubility and the dissociation of the ion are enhanced due to the nanoparticle size of ACC, which increases the surface area by up to 1 × 10^6^ [26].

## 2. Materials and Methods

All animal experiments were approved by the Israeli Animal Testing Council. The registry numbers of approvals are as follows: IL-18-2-27, IL-16-01-131 (for LLC experiments) and IL-19-09-404 (for xenograft experiments).

All animals were purchased from Envigo, Rehovot, Israel. Experiments started after 5 days of acclimation. Mice were housed using 12 h light/dark cycles and had water and chaw ad libitum.

All solutions, reagents, and LLC and A549 cell lines were purchased from Biological Industries, Bet-Haemek, Israel, unless otherwise mentioned.

Dose selection: Preliminary studies of the LLC model have been conducted for selecting the appropriate dosage for the study. Different ACC concentrations were assessed (0.1, 0.3 and 0.5% elemental calcium in freshly prepared ACC suspensions) given via IP injections. The effect on the tumors’ deceleration growth rates was most profound with 0.5% elemental calcium; therefore, we continued to larger experiments with this dose regimen. As for cisplatin dose, the selected dose is within the acceptable range for mice models [41].

### 2.1. ACC Preparation Protocol

Stabilized ACC suspensions (with 3 mol% of sodium tripolyphosphate (STTP) as a stabilizer) were used for the studies. Fresh and stable suspensions were prepared immediately before the experiments according to a proprietary procedure, and the concentration of their calcium content was calculated. The suspension used in the experiments consisted of 1.22% ACC (containing 0.5% elemental calcium).

### 2.2. Evaluations of Various ACC and CCC Suspensions on Acidified Medium with Serum

This experiment evaluates the ability of ACC and CCC to affect the pH of a medium supplemented with 10% (*v*/*v*) serum. The purpose of this experiment was to achieve a mildly acidic environment similar to that found in tumors [42] by adding lactic acid. Once the acidic environment was achieved, ACC or CCC were added, and the pH was measured. Lactic acid was chosen since it is naturally found in the body in its conjugated base form and is associated with acidosis as the product of glycoysis. A total of 2 mL of fetal bovine serum (FBS) was added to 18 mL of DMEM/F12 medium. The solution was placed inside a sterile tissue-sample cup, and a hole was made at the top of the cup into which a pH probe was inserted. The cup was placed on a magnetic stirrer (JB-10 stirrer, Inesa, Shanghai, China), and the solution was constantly stirred during the measurement. A pH meter (MesuLab, PXSJ-216F ion meter, MRC, Holon, Israel) was connected to a PC, and the continuous data-logging of pH measurements was performed using REXDC2.0 software. After the system was set and the pH measurement and data logging were started, 20.5 µL of lactic acid solution (final concentration was 4.7 mM) was added to the solution to slightly reduce the pH acidotic stage of about 6.7, similar to the pH found in many solid tumors [42]. Once the stabilization of the acidic pH was achieved, the following samples were added, each having a volume of 3 mL: (1) freshly prepared ACC suspension, (2) ACC in powder form resuspended in distilled water (4% *w/v*), and (3) CCC in powder form resuspended in distilled water (4% *w/v*). This experiment was repeated with suspensions and prepared differently each time based on proprietary procedures. The measurement duration was approximately 470 s (about 7.8 min), sufficient time to observe that the change in pH was stable.

### 2.3. Amorphous Phase Validation of ACC and Quantification via X-ray Diffraction (XRD) Analysis

A calibrated XRD method was used for determining the amorphous phase percentage in the ACC product as synthesized and as dried or suspended forms after storage periods. The quantitative percentage of the amorphous phase was calibrated by concentration curves that were compared at different mixing ratios of pure ACC and the calcite and vaterite crystalline phases. This analytical technique was performed for each ACC suspension used in the study.

### 2.4. Scanning Electron Microscopy (SEM) of ACC

An ACC suspension stabilized with STTP was filtered and gently dried in steps that were designed to prevent any crystallization and characterized by scanning electron microscopy (SEM). It is apparent that ACC is morphologically different from the typical crystalline calcium carbonate, which is cubic and within the micron range at tens of microns. The SEM analysis of ACC revealed that the microstructure of dry particles is composed of aggregated primary nanoparticles within the range of 10 to 100 nm compared to the micrometric calcite particles obtained during the same synthesis method, wherein the drying process malfunctioned.

### 2.5. In Vitro Culture of LLC and A549 Cell Lines

Cells were cultured in a humidified incubator at 37 °C and 5% CO_2_ in T-25 flasks (Corning^®^ Merk, Rehovot, Israel). The culture medium comprised 90% Dulbecco’s Modified Eagle Medium/Nutrient Mixture F-12 (DMEM-F12), 10% (*v*/*v*) fetal bovine serum (FBS), 2 mM glutamine, Penicillin G Sodium Salt: 10,000 units/mL, streptomycin sulfate: 10 mg/mL (Pen/Strep). For A549 cells cultured in the presence of 2 mM (elemental calcium) ACC, an additional experiment evaluating how different ACC concentrations affect A549 cells growth was performed. A549 cells were cultured in the presence of 1, 2 and 4 mM of elemental calcium in the form of ACC suspensions in DMED-F12, calcium-depleted medium. A DMEM-F12 full medium (containing the standard calcium level) was used as a control. All media were supplemented with 10% (*v*/*v*) FBS, 2 mM glutamine, Penicillin G Sodium Salt: 10,000 units/mL, streptomycin sulfate: 10 mg/mL (Pen/Strep). This experiment was done in quadruplets for each concentration, and each well was counted twice using a hemocytometer. All cells were seeded at a concentration of 2 × 10^4^ cells per mL, and cells were counted on days 1, 2, 4, 5 and 6 post-seeding.

### 2.6. In Vivo Experiments with Lewis Lung Carcinoma Cells

Freshly harvested LLC cells in a concentration of 2.6 × 10^5^ in 100 μL phosphate-buffered saline (PBS) were subcutaneously injected into the right flank of C57BL/6 female mice aged 5–7 weeks under the light anesthesia of the mice achieved by isoflurane inhalation. Once the tumor reached a volume of >50 mm^3^ on day 12, the study mice were allocated randomly into study groups (n = 8, per group), and treatments were given accordingly (day 0 is the day of the LLC injection). Mice that did not develop a tumor within this size range were excluded from the study. The study groups were as follows: (1) a group receiving the vehicle (1.5% (*w*/*v*) NaCl) as a negative control; (2) cisplatin 3.3 mg/kg (Pharmachemie B.V. (Teva Group), Haarlem, The Netherlands) (treatment was given according to manufacturer instructions every 3 days) as a positive control; (3) ACC suspension (Amorphical, Nes-Ziona, Israel) at a concentration of 1.22% (*w*/*v*) ACC (contains 0.5% elemental calcium), which corresponds to a dose of 0.244 g/Kg ACC for a mouse that weighs 20 g; (4) ACC and cisplatin combined at the above doses. ACC and the vehicle were administered via intraperitoneal (IP) injections twice a day at a volume of 200 µL for each injection. Cisplatin was given via IP injections twice a week according to manufacturer instructions.

The study duration was 26 days, including 14 days of treatments. The tumor growth was measured approximately every other day using a digital caliper, and the tumor volume was calculated according to the following equation: Volume = (Length × Width^2^)/2, where length represents the largest tumor diameter, and width represents the smallest tumor diameter. At the end of the study, the mice were euthanized, and the tumors were removed for the cathepsin B activity assay.

### 2.7. Cathepsin B Activity Measurements

Mice with subcutaneous LLC tumors, which were treated with the vehicle, ACC, and cisplatin, were resected after the study’s termination and animal euthanasia (n = 8, per group). All conditions of maintenance and handling were similar between the groups.

For the assay, 20 mg of each extracted tumor was weighed and lysed. Measurements were performed using the Cathepsin B Activity Assay Kit (Fluorometric) (ab65300 by Abcam, Cambridge, UK). The assays were performed on tumors from three groups, excluding the group that received the combined cisplatin and ACC treatment due to a low tumor weight. The lysed samples were treated per the kit’s instructions. In brief, 50 μL of lysis buffer was used, and the cells were incubated on ice for 10 min. Centrifugation was carried out at 20,000× *g* for 5 min, and then the supernatant was transferred to a new tube. In total, 50 μL of the lysate was added to an opaque black 96-well plate. Then, 50 μL of the reaction buffer and 2 μL of the 10 mM substrate Ac-RR-AFC were added to each sample. In this study, 2 μL of the inhibitor was used as the negative control. The Infinite^®^200Pro (Tecan, Männedorf, Switzerland) microplate reader was used with 400 nm excitation and a 505 nm emission filter to analyze the fluorescence intensity for cathepsin B enzymatic activities after incubating the samples at 37 °C for 1 h in the dark.

### 2.8. Xenograft Model of A549 Human NSCLC

Two cohorts of this study were performed. Female athymic nude mice, 5–7 weeks old, were used for this experiment. After acclimation, animals were subcutaneously injected with 100 µL of human lung cancer cell line A549 suspended in ice-cold phosphate-buffered saline (PBS) having a cell concentration of 5 × 10^6^ cells /mL, and the injections were performed in the right flank of the mice. The injection day was considered “Day 0” of the experiment. Once tumors reached a measurable size larger than 40 mm^3^, the mice were randomly allocated into 2 treatment groups: ACC suspension (same concentrations as described in Section 2.6 above) or saline (the byproduct of ACC is NaCl). Both saline and ACC were administered via IP injections of 200 µL twice a day. Tumor growth was measured approximately every 3 days. In this study, the first cohort consisted of 12 animals per group (total of 24 animals), and treatment duration was 22 days; the second cohort consisted of 7 animals per group (total of 14 animals) and a treatment duration of 20 days.

### 2.9. Differential Gene Expression Evaluations of ACC-Treated A549 Cells

The A549 cell line was cultured as described above in Section 2.1. Cells were divided into 2 groups: either cultured in the DMEM-F12 regular medium (containing 1.1 mM calcium chloride (CaCl_2_)) or the DMEM-F12 medium without calcium to which 2 mM of ACC had been added. Both groups were cultured in triplicate and for 8 passages, after which their RNA was isolated using the Promega SV Total RNA Isolation System (Promega Corp., Madison, WI, USA). This procedure was carried out for the cells of both groups in triplicate.

To avoid batch effects that could interfere with the signal in the experiment, the extraction from all nine samples was performed on the same day and at the same time using the same kits, persons, etc. The RNA concentration was detected for each sample (Nanodrop^®^ spectrophotometer, Thermo Fisher Scientific, Waltham, MA, USA) and the concentration was determined by TapeStation (Agilent, CA, USA).

RNA-seq libraries were prepared at the Crown Genomics Institute of the Nancy and Stephen Grand Israel National Center for Personalized Medicine, Weizmann Institute of Science. Libraries were prepared using an inhouse mRNA-seq protocol. Briefly, the polyA fraction (mRNA) was purified from 500 ng of total input RNA, followed by fragmentation and the generation of double-stranded cDNA. After Agencourt Ampure XP beads cleanup (Beckman Coulter, CA, USA) and end repair, base addition, adapter ligation, and PCR amplification steps were performed. Libraries were quantified by Qubit (Thermo Fisher Scientific, MA, USA) and TapeStation (Agilent, CA, USA). Sequencing was done on a NextSeq instrument (Illumina, CA, USA), allocating approximately 29 M reads per sample (single read sequencing, 84 bases).

Poly-A/T stretches and Illumina adapters were trimmed from the reads using Cutadapt; resulting reads shorter than 30 bp were discarded. Reads were mapped to the Homo Sapiens GRCh38 reference genome using STAR (version 2.4.2a), supplied with gene annotations downloaded from Ensembl (release 92). The alignEndsType was set to EndToEnd, and outFilterMismatchNoverLmax was set to 0.04. Expression levels for each gene were quantified using htseq-count using the gtf above. Differential expression analysis was performed using DESeq2 (version 1.10.1). The betaPrior, Cook’s distance cutoff, and independent filtering parameters were set to False. Raw *p* values were adjusted for multiple testing using the procedure of Benjamini and Hochberg [43]. We defined significantly differentially expressed genes as those with FDR ≤ 0.05, absolute fold change ≥ 2, and a count of at least 30 in one of the samples. Ingenuity^®^ Pathway Analysis (IPA^®^) software (Qiagen, Hilden, Germany) was used for enrichment analysis, and a Z-score above 2 or below −2 was considered significant.

The RNA-Seq data discussed in this publication have been deposited in NCBI’s Gene Expression Omnibus [44] and are accessible through GEO Series accession number GSE235316 (https://www.ncbi.nlm.nih.gov/geo/query/acc.cgi?acc= GSE235316) accessed on 21 June 2023.

Gene set enrichment analysis (GSEA) was performed on a pre-ranked dataset sorted by log2FC (ACC versus untreated) using the GSEA 4.2.2 desktop application [45] with the Hallmark gene sets from the Molecular Signature Database v2023 [46]. Only genes that had a count of at least 50 in at least one of the samples were considered.

### 2.10. Statistics

Values are expressed as the mean ± SEM. For evaluating whether different treatments affected tumor growth rates, we compared the differences in the tumor volume of each treatment group on each measurement day. For in vivo LLC and cathepsin B and in vitro A549 proliferation evaluations, the results were compared by ANOVA, followed by Tukey’s multiple comparison test. The significance value was set at *p* < 0.05. For A549 xenograft studies, the difference in the tumor volume in the treatment groups was analyzed by an unpaired two-tailed Student’s *t*-test to determine the differences between the two groups for each measurement time point. Significance was set at *p* < 0.05.

## 3. Results

### 3.1. ACC and CCC Evaluation in an Acidified Medium with Serum

**Figure 1:**
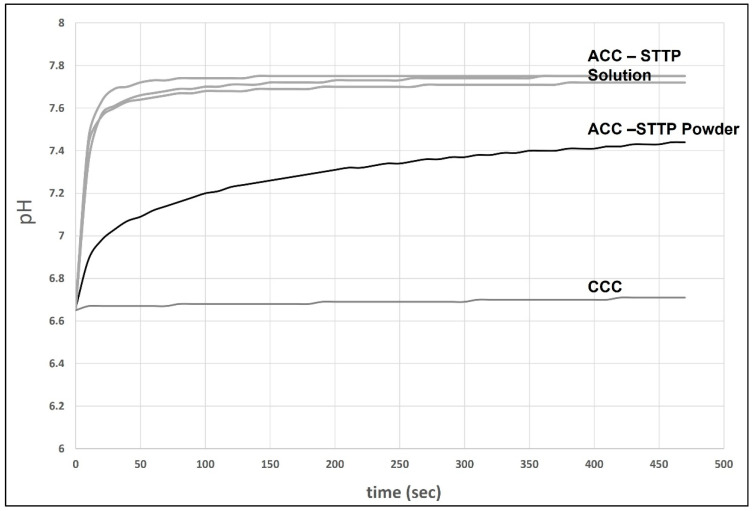
ACC effect on pH. Medium pH change after adding the following solutions: (1) ACC stabilized with STTP, a freshly prepared suspension, similar to the suspensions used in these experiments (repeated 3 times). (2) Solid dried ACC powder that was resuspended in water (4% *w*/*v*). (3) CCC powder that was resuspended in water (4% *w*/*v*).

In this experiment, we wanted to evaluate the effects of different forms of calcium carbonate, either amorphous (ACC) or crystalline (CCC), to elevate the medium’s pH once it was acidified. We chose to reduce the medium’s pH with lactic acid since it resembles the physiological conditions of acidosis more. The results of Figure 1 show that the ACC solution (similarly to the solutions used in the in vivo and in vitro experiments described here) or those dispersed in water immediately elevated the medium’s pH from about 6.7 to 7.7 and 7.4, respectively, and the pH was stabilized at these basic levels. However, CCC was unable to change the pH of the medium due to its very high insolubility at pH levels above 6. There was a slight increase in the medium’s pH after CCC was added, and this was most likely due to the buffering capacity of the DMEM/F12 medium.

### 3.2. Amorphous Phase Validation of ACC by XRD Diffractograms

**Figure 2:**
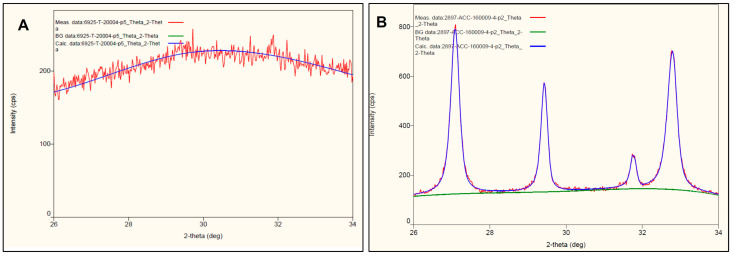
XRD diffractograms of (**A**) completely amorphous ACC and (**B**) the partially crystallized one (the blue line picks) in the angle range from 26 to 34.

Below is a representative figure (Figure 2) that reveals the significant difference of XRD diffractograms within the range of 26 to 34 degrees with respect to ACC (Figure 2A) and the partially crystallized batch containing nanometric calcium carbonate (calcite) obtained when the synthesis process was inadequate (Figure 2B) (the red lines). The lack of patterns beyond the noise level in Figure 2A indicates 100% of the amorphous phase. The very wide pattern assigned to the XRD of calcite in Figure 2B indicates that calcite particles are also nanometric in this specific case. Sharper and narrower patterns mean large crystalline particles.

### 3.3. SEM of ACC

**Figure 3:**
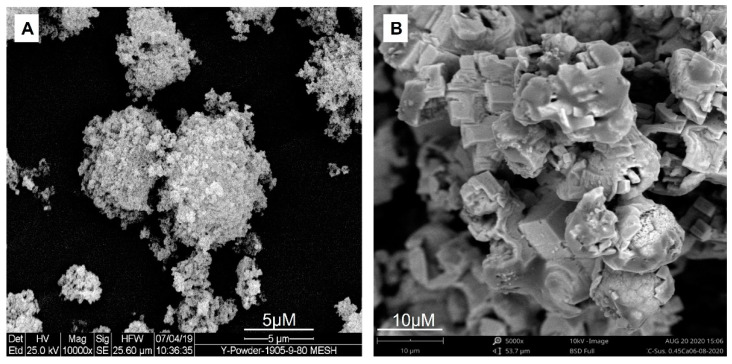
Scanning electron microscopy representative images of (**A**) stabilized ACC and (**B**) ACC that was crystallized when the processing line malfunctioned.

Figure 3A illustrates the microstructure and morphology of a pure ACC compared to an ACC that was unintentionally crystallized (Figure 3B) during the drying of amorphous nanoparticles. Notice that the morphology in this case is completely different from the natural cubic form of calcite when it crystalizes in an aqueous environment.

### 3.4. ACC Effect on A549 Proliferation

**Figure 4:**
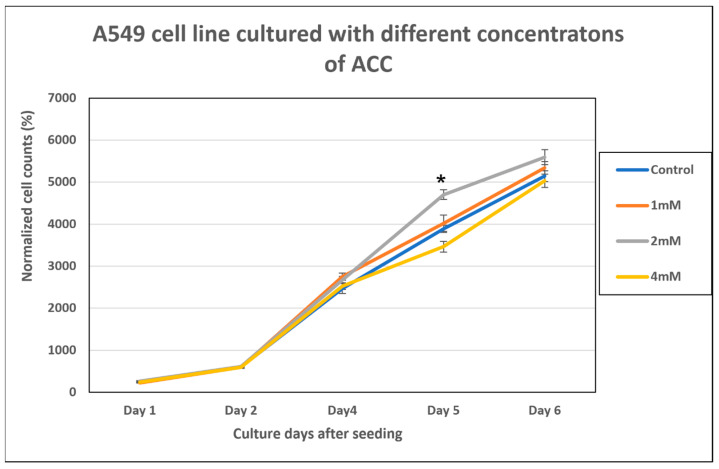
Cell counts of A549 cells cultured with 1, 2, and 4 mM elemental calcium in ACC suspensions, compared to the control (regular DMEM-F12 medium). The graph is presented as the normalized percentages, compared to seeding day, which constitutes 100%. Data are presented as mean ± SEM. * This is a statistically significant result relative to the control and other groups (*p* < 0.05).

In this experiment, A549 cells cultured with different concentrations of ACC (1, 2 and 4 mM elemental calcium in ACC suspensions) were added to the DMEM-F12 calcium-depleted medium. Cells cultured in the DMEM-F12 full medium served as the control. Figure 4 below summarizes the results. We can see that, overall, no statistical significance was detected among groups in the cell counts on each day. Only on Day 5 post-seeding, the 2 mM concentration was significantly higher than the control. Interestingly, even 4 mM of ACC did not show a negative effect for these cells.

### 3.5. ACC Effect on LLC Tumor Growth Rates

**Figure 5:**
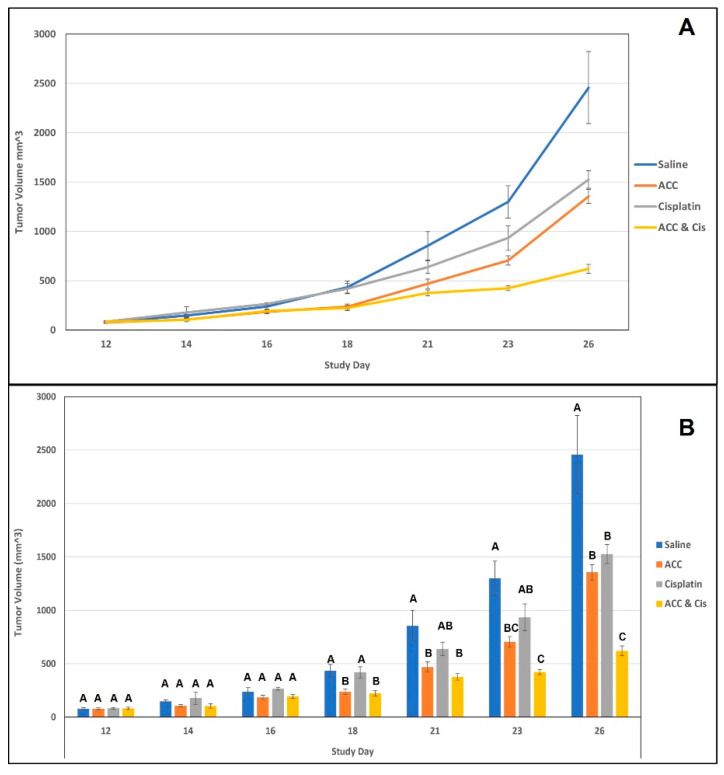
This figure shows the tumor volumes of each treatment group during the treatment days of the experiment (days 12 to 26). Data are presented as mean ± SEM. (**A**) presents the tumor volume as a continues change throughout the treatment days. (**B**) shows the same results but with the statistical analysis done for each measurement day. Statistical significances are represented by different letters (*p* < 0.05). N = 8, per group.

In this experiment, we evaluated tumor growth rates in an LLC subcutaneous model (see Figure 5 below). Four different treatments were carried out once tumors reached a measurable size: ACC, cisplatin, vehicle, and ACC combined with cisplatin. The treatments were carried out for 14 consecutive days (from day 12 until day 26). The results demonstrated that ACC treatment reduced tumor growth rates in a similar manner to that of cisplatin, with ACC reducing the tumor volume in 44.83% and cisplatin in 37.87% on day 26 in a statistically significant manner compared to the vehicle (Figure 5B). ACC’s effect on the reduction in tumor volume was observed on day 18 (after 6 days of treatment) onwards (Figure 5, orange). Similarly, the combined treatment of ACC and cisplatin (Figure 5, yellow) is also apparent from day 18 (after 6 days of treatment), whereas cisplatin’s effect on tumor deceleration was significantly reduced only on the last day of the study on day 26 (after 14 days of treatment) (Figure 5, gray). The combined treatment of ACC and cisplatin resulted in a highly increased anti-tumor effect showing a reduction of 74.75% in the tumor volume compared to the vehicle on day 26, which was statistically significant from all other treatments (Figure 5B).

### 3.6. ACC Effect on Cathepsin B Activity

After the mice with LLC tumors were euthanized on day 26, the tumors were resected from the animals, and cathepsin B activity was measured. Cathepsin B activity was significantly lower in tumors resected from mice treated with ACC and cisplatin compared to vehicle-treated animals. This observation supports our hypothesis of the solid-base buffering effect of the ACC since cathepsin B activity is known to increase in acidic environments and is associated with the tumor’s aggressiveness [16,17,19]. Tumors from the combined ACC and cisplatin treatment group were not analyzed for cathepsin B activity.

**Figure 6:**
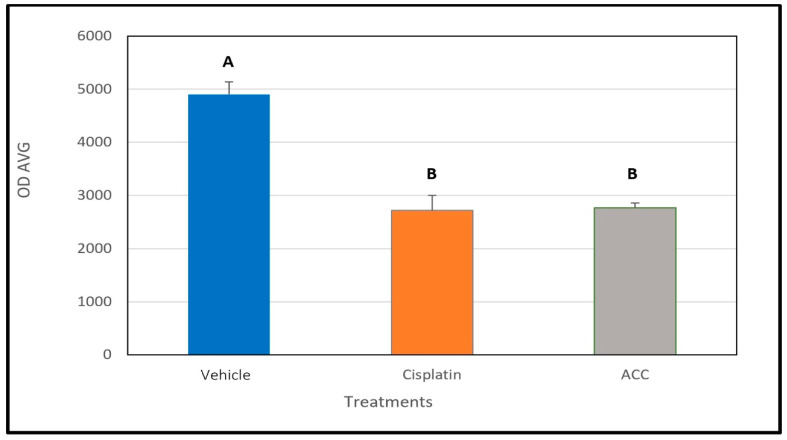
The effect of the different treatments on cathepsin B activity in tumors resected on day 26 (n = 8, per group). Data are presented as the mean ± SEM. Different letters represent a statistical significance of *p* < 0.05.

The results shown in Figure 6 revealed that samples taken from tumors of ACC and cisplatin-treated animals had significantly lower cathepsin B activity levels compared to tumor samples from vehicle-treated animals.

### 3.7. ACC Effect on Human A549 NSCLC Xenograft Growth Rates

**Figure 7:**
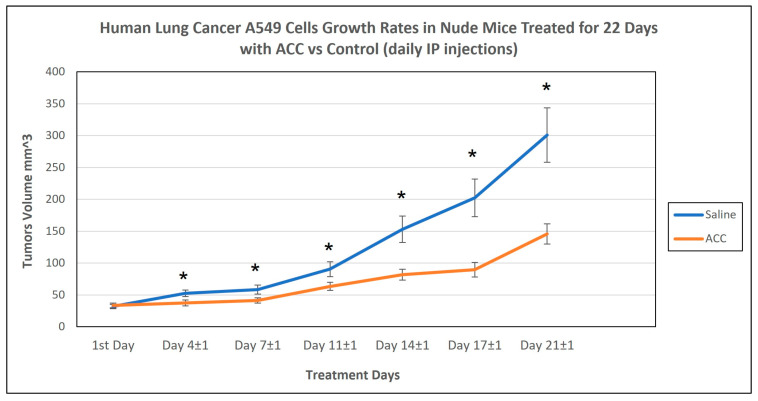
Xenograft model of A549 NSCLC human lung cancer tumor model transplanted subcutaneously into nude mice. The graph represents the combined results of two experimental cohorts. Treatments of ACC or vehicle were given daily via IP injections, and n = 19 per group. Data are presented as the mean ± SEM. For each measurement day, Student’s *t*-test analysis was performed. The results were statistically significant from day 4 ± 1 onwards, * representing a statistical significance between the treatment groups (*p* < 0.05).

In these experiments (2 cohorts with a total number of 38 animals (19 mice per group)), we wanted to evaluate whether ACC can decelerate tumor growth rates in a human non-small cell lung carcinoma model using the A549 cell line. We established a subcutaneous xenograft model and compared ACC and vehicle (saline) treatments. The results, shown below in Figure 7, reveal that ACC decelerated tumor growth rates significantly compared to the control. On the last treatment day, ACC-treated mice had an average tumor volume of 145.6 mm^3^, and control-treated animals had an average tumor volume of 300.9 mm^3^. This observation reflects a reduction of 51.6% in tumor volume with the ACC treatment compared to the control.

### 3.8. ACC Effect on the Differential Gene Expression of A549 Cells

**Figure 8:**
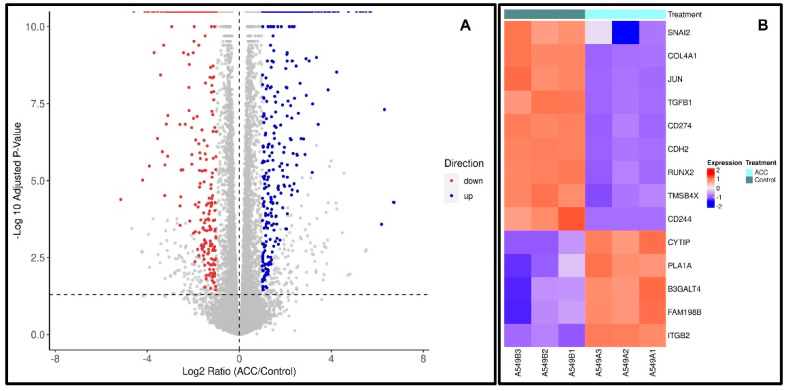
A549 lung cancer cells’ differential gene expression, evaluated for ACC-treated cells versus non-treated cells. (**A**) depicts a volcano plot, wherein each spot indicates a gene and its log2-fold change vs. log10-adjusted *p*-value. Light blue dots indicate genes that were significantly downregulated. Red dots indicate genes that were significantly upregulated. Gray dots indicate all other genes. (**B**) is a heatmap representation of selected differentially expressed genes of interest (a total of 14 genes). The log2-normalized counts were standardized to have a zero mean and standard unit variance for each gene. The expression profile is accompanied by a color bar indicating the standardized log2-normalized counts. It is important to note that two of the genes of interest had low counts but are still included here.

To follow the effect of ACC on the gene-expression profile of cells, A549 cells were cultured either with or without ACC for eight passages. Cells were collected and subjected to RNA-seq analyses. Differential gene-expression analyses revealed that 546 genes were upregulated, and 432 genes were downregulated following the ACC treatment, resulting in a total of 978 genes that were differentially expressed. Figure 8A,B illustrate these results visually using a volcano plot diagram (Figure 8A), which depicts genes that were statistically up- or downregulated. Moreover, the influence of the differential gene expression was evaluated using Ingenuity Pathway Analysis (IPA), and the prediction of these changes demonstrates mostly changes in tumorigenic pathways (Table 1). The full IPA results can be found in the Supplementary Material of this publication. Overall, these results suggest that ACC changed A549 lung cancer tumor cells towards an anticarcinogenic phenotype and increased the immune system activity pathways. Interestingly, the homeostasis pathway is predicted to be increased (Table 2).

**Figure 9:**
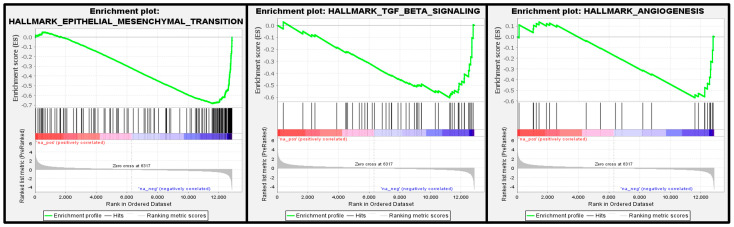
Gene-set enrichment analysis (GSEA) of selected pathways. FDR q-values were: 0.0 for epithelia mesenchymal transition; 0.020316496 for TGFβ signaling; 0.020316496 for the angiogenesis pathways.

Results from the GSEA seen in Figure 9 show three major cancerous pathways that were downregulated, the EMT, TGFβ and angiogenesis pathways.

## 4. Discussion

In this study, we used a stable form of ACC as observed by XRD (Figure 2), which is composed of primary particles in the nanometric range (40–100 nm) observed in SEM imaging (Figure 3). The ACC solution used in these experiments was able to elevate the pH of the acidified medium with 10% serum, whereas CCC was not able to elevate the pH due to its very poor solubility in mild acidic pH conditions (Figure 1). This improved solubility of ACC compared to crystalline calcium carbonate was demonstrated in previous studies in which it also resulted in improved bioavailability [25,26,27,28].

The treatments in the experiments described here were carried out via IP injections as a systemic route of administration. Moreover, injections were given twice daily to maintain an intensive drug regimen, taking into account that changes in the tumor’s pHe may be transient in nature. This transient elevation of tumor microenvironment pH was detected in a study where nanosized vaterite was intravenously administered to mice in a breast cancer model [67].

The ACC effect on the tumor growth rate was evaluated in two in vivo models of lung cancer: (a) LLC subcutaneous model and (b) A549 subcutaneous xenograft model. ACC produced a significant effect in both models compared to the control (vehicle-treated animals) and decelerated tumor growth rates, as illustrated in Figure 5 and Figure 7. The effect of ACC in the LLC model was similar to that of the positive control animals treated with cisplatin, the long-established chemotherapy drug (as shown in Figure 4 and Figure 5). The most profound effect was observed when cisplatin and ACC treatments were combined. This combination treatment resulted in the lowest growth rate of tumors compared to other treatments with a reduction of 74.75% in tumor volume compared to vehicle (in a statistically significant manner) and may indicate a synergetic effect.

When the activity of cathepsin B in LLC tumors resected from mice treated with vehicle, ACC, and cisplatin is examined, a significantly lower activity was determined for ACC and cisplatin-treated tumors compared to those in vehicle-treated mice (Figure 6). These results support our hypothesis that ACC has an antitumor effect because it elevates the pHe and maintains the base-buffering effect in the tumor microenvironment throughout ACC administration, resulting in less cathepsin B production. Cathepsin B is active in an acidic environment and is associated with poor prognosis and the increased aggressiveness of cancer [15,16,17]. Moreover, cathepsin B levels in the TME are associated with the effect tumor cells have on non-malignant cells in the TME; i.e., their reaction to the acidic environment causes the release of cathepsin B into the intercellular area [15]. Most likely, the reduction in cathepsin B activity observed in cisplatin-treated tumors is a result of the cytotoxic effect on cancer cells, leading to a lower release of cathepsin B by the cells [15]. However, ACC is not a cytotoxic compound, as seen in Figure 4, when added to cancer cells. In vitro normal growth is observed similar to that of the culture medium without ACC. Other studies with ACC and vaterite, either for drug delivery [30] or given as a treatment [36,65], emphasize its safety. Thus, ACC’s therapeutic effect is most likely attributed to the cumulative effects caused by elevating the pHe in the TME, such as the following: (a) lower metastases [7,8,9,10,16,31,32,33,68,69,70]; (b) lower proliferation rates [7,10,14,71]; (c) reduced cathepsin B and other protease activities [15]; (d) increased immune-system ability to react in the tumor environment [21,72]; and (e) decreased chemoresistance [73,74]. The enhanced effect observed in decelerating tumor growth rates when ACC was combined with the cisplatin treatment may indicate decreased chemoresistance or an additive effect of the two compounds. Cisplatin changes the tumor cell’s metabolism and lowers its glycolytic metabolism, which is accompanied by increased oxphos [75,76]. This metabolic shift in cisplatin-treated animals might be another reason for the reduced cathepsin B activity observed in Figure 6. A similar increase in oxphos activity and reduced glycolysis was observed in a “Seahorse Test” that was comparatively performed on the 4T1 murine breast cancer cell line in the presence and absence of ACC (unpublished results). Som et al. [67] observed a similar metabolic shift in HT1080 fibrosarcoma-bearing mice in which glucose uptake was reduced after injection of nano-vaterite [67].

As for the enhanced combination treatment, elevating the pHe by ACC possibly enhances the cisplatin anti-proliferating effect on tumor cells and enhances the shift in metabolism towards oxphos, resulting in lower tumorigenicity. A recent study showed that carbonate ions have no effect on cisplatin’s binding to DNA [77], strengthening the hypothesis that this combined effect of ACC and cisplatin is due to the elevation of pHe by carbonate ions.

An additional explanation for the enhanced effect of the combined treatment might be attributed to a drug-delivery effect. The ACC nanometric structure contains porosity and has a significant surface area (in the range of 40 to 60 m^2^/g), resulting in the high absorption/adsorption capability of molecules such as cisplatin [29]. The half-life of cisplatin is in the range of days in repeated administration [41,78]. It is plausible that ACC particles have encountered cisplatin and created a “loaded” complex. If this has occurred, then once this cisplatin-loaded ACC compound reached the tumor’s acidic environment, an increased local release of the drug was achieved, enhancing the antitumor effect.

The xenograft experiments revealed a similar pattern of decelerating tumor growth rates (as illustrated in Figure 7) for ACC-treated animals compared to the control in a significant manner. The average final volume of ACC-treated mice was 145.6 mm^3^ (Figure 7), which is less than half the size of the average volume of the control group, which was 300.9 mm^3^, amounting to a reduction of 51.6% in tumor volume.

It is well established that cancer cells undergo genetic and epigenetic changes that support their multifaceted cancerous phenotypes [79,80,81,82]. In this study, we evaluated the differential gene expression of an A549 cell line, a human NSCLC line, that was cultured with or without ACC in the medium. There were profound alterations in gene expression patterns (Figure 8). The analysis of the genes’ up- or downregulation differences and their potential involvement in cancer progression (as detailed in Table 1 and Table 2) reveal a general antitumorigenic effect. Some of the altered genes are already serving as treatment targets, e.g., PDL-1, ITBG2, TGFB1, and c-JUN, in many cancer types, including lung cancer [48,49,50,51,52,53,54,55,56,57,58,59,60,61,62,63,64,65,66,67,68,69,70,71,72,73,74,75,76,77,78,79,80,81,82]. Moreover, GSEA analysis showed that the down-regulated genes were enriched in three major precancerous pathways: EMT, TGFβ and angiogenesis. These pathways are associated with acidosis in TME. Although we cannot directly deduce protein functions from the expression of genes, the described results combined with the xenograft’s results indicate that ACC alters cancer cell gene expression to produce anti-tumorigenic effects and, together with the modulation of the acidic tumor microenvironment, results in the in vivo deceleration of tumors. Additionally, downregulated SNAI2/SLUG genes induce angiogenesis, and downregulated JUN indicates a loss of E-cadherin, and reduced cadehrin-2 suggests a reduced invasion capability [52,59,61,63,64].

New and safe treatments for lung cancer are still being researched, especially those that can be given in combinations. The results presented here, although preliminary, present the anti-cancer activity of ACC. Although direct evidence is not yet established, the known properties of ACC (i.e., increased solubility in mild acidic conditions and bioavailability) support the hypothesis that these anti-tumorigenic effects are due to modulation of the pHe in the TME. Moreover, the enhanced effect of the combined ACC and chemotherapy (cisplatin) is very promising for further developing ACC as an anticancer treatment.

The main limitation of this study is the lack of direct evidence for changes in tumor pHe. This study revealed efficacy in treating cancer, which is associated with TME acidosis, but more direct evidence is still needed. In addition, we did not evaluate metastasis.

Further research progression is needed for understanding ACC biomedical mechanisms, its pharmacokinetics as a function of administration modes, and the evolution of its improved formulations (oral and inhalation).

## 5. Conclusions

In conclusion, ACC has demonstrated efficacy in reducing tumor growth rates in vivo in two NSCLS models (LLC and A549 xenograft). ACC has also reduced cathepsin B activity in LLC tumors and has altered the gene expression of the A549 cell line toward an anti-tumorigenic phenotype. All the study outcomes are associated with the tumor’s acidic microenvironment and its modulation. These results, combined with the substantial safety of ACC, strongly support the rationale for further accelerating the clinical evaluation of ACC as an anti-malignancy substance.

## 6. Patents

ACC synthesis and uses are protected by several patents. The most relevant patents for this paper are as follows: US 14/744,726; WO 2017/125917; WO 2021/181372.

## Figures and Tables

**Table 1:** The influence of the differential expression of genes in A549 lung cancer cells and ACC-treated cells versus non-treated cells on different pathways.

Diseases or Functions	Predicted Activation	Activation z-Score
Glucose metabolism disorder	Decreased	−2.866
Lymphoreticular neoplasm	Decreased	−2.627
Cancer of cells	Decreased	−2.543
Dysglycemia	Decreased	−2.441
Development of digestive organ tumor	Decreased	−2.304
Migration of smooth muscle cells	Decreased	−2.258
Cell movement of smooth muscle cells	Decreased	−2.218
Tumorigenesis of epithelial neoplasm	Decreased	−2.177
Lymphohematopoietic cancer	Decreased	−2.174
Hematologic cancer	Decreased	−2.174
Myeloid or lymphoid neoplasm	Decreased	−2.167
Neoplasia of blood cells	Decreased	−2.164
Growth of smooth muscle	Decreased	−2.158
Synthesis of glycosaminoglycan	Decreased	−2.154
Formation of solid tumor	Decreased	−2.153
Liver lesion	Decreased	−2.152
Hematological or lymphatic system tumor	Decreased	−2.095
Hematologic cancer of cells	Decreased	−2.064
Proliferation of smooth muscle cells	Decreased	−2.037
Lymphoma	Decreased	−2.011
Lymphatic system tumor	Decreased	−2.004
Immune mediated inflammatory disease	Increased	2.031
Cell death of immune cells	Increased	2.11
Differentiation of mononuclear leukocytes	Increased	2.117
Breast or ovarian carcinoma	Increased	2.178
Breast cancer	Increased	2.178
Acute lung injury	Increased	2.188
Transport of metal	Increased	2.214
Transport of metal ion	Increased	2.322
Relaxation of muscle	Increased	2.348
Breast or ovarian cancer	Increased	2.373
Breast or gastric cancer	Increased	2.373
Breast or gynecological cancer	Increased	2.373
Lung injury	Increased	2.386
Transport of monovalent inorganic cation	Increased	2.433
Transport of inorganic cation	Increased	2.496
Multiple cancers	Increased	2.556
Breast or pancreatic cancer	Increased	2.557
Secretion of molecule	Increased	2.581
Leukopoiesis	Increased	2.654
Cellular homeostasis	Increased	2.712
Transport of molecule	Increased	3.239

**Table 2:** A summary of selected genes that were statistically differentially expressed in ACC-treated cells compared to untreated cells and their possible antitumorigenic role. It is important to note that 2 of the genes of interest had low counts but are still included here. The genes are SNAI2 and CD244, which had low counts of 19 and 12, respectively.

Gene Name (Encoded Protein)	ACC Effect on Gene Expression Fold Change (*p* Value; *p* adj)	Potential Outcome Meaning and Relevant References
CD274 (PDL-1)	−3.8 (0; 0)	Activation of the immune response [47,48,49]
CYTIP (Cytohesin 1-Interacting Protein)	+72.5 (3.75 × 10^−5^; 2.62 × 10^−4^)	Activation of the immune response of T cells [50].
ITGB2 (CD18)	+7.2 (0; 0)	ITGB2 overexpression inhibited the proliferation, migration, and invasion of NSCLC cell lines [51].
JUN (Protooncogene JUN)	−3.6 (0; 0)	Inhibition of c-JUN decreased angiogenesis in A549 cells in vivo and in vitro [52].
RUNX2 (RUNX2 or CBF- alpha-1)	−15.35 (0; 0)	RUNX2 was overexpressed in the tissues of patient with primary NSCLC and lung metastasis. Moreover, overexpression observed when epithelial–mesenchymal transition (EMT) increased. Additionally, absence of RUNX2 decreased EMT and invasion capacity in A549 cells [53].
CD244/2B4 (CD244)	−227.54 (0.00441; 0.0191)	CD244 is an immunomodulator of T-cells and NK cells. It is associated with an immunosuppressive environment in cancer [54].
TGFB1-(TGF β)	−1.6 (0; 0)	TGF-β1 signaling is a potent inducer of the EMT in various types of cancer, including NSCLC [55,56] and specifically in A549 cells [57].
TMSB4X (Thymosin beta4)	−1.67 (0; 0)	Upregulation of TMSB4X was found to be associated with chemoresistance in lung cancer tissues [58].
SNAI2/SLUG-(Snail Family Transcriptional Repressor 2)	−3.758 (0.0031; 0.014)	Overexpression of SNAI2 is associated with EMT and poor prognosis of cancer patients [59] and with loss of E-cadherin (which is associated with increased infiltration and invasiveness) [60].
COL4A1 (collagen type IV alpha chain 1)	−2.14 (0; 0)	Overexpression is associated with increased proliferation and migration and poor prognosis in several cancers [61,62]
CDH2 (Cadherin-2)	−1.42 (0; 0)	High expression is associated with angiogenesis promotion and poor survival in lung cancer [63], as well as with increased likelihood of brain metastasis [64].
PLA1A (phosphatidylserine-specific phospholipase A1)	+3.43 (1.29 × 10^−8^; 1.5 × 10^−7^)	Overexpression has been found to limit aggressiveness in lung adenocarcinoma [65]
B3GALT4	+1.42 (10^−10^; 1.3 × 10^−9^)	Overexpression associated with remodeling of TME and enhancing immunotherapy efficacy [66]

## Data Availability

The RNA-Seq data discussed in this publication have been deposited in NCBI’s Gene Expression Omnibus [44] and are accessible through GEO Series accession number GSE235316 (https://www.ncbi.nlm.nih.gov/geo/query/acc.cgi?acc= GSE235316). Accessed on 21 June 2023.

## Supplementary Materials

The following supporting information can be downloaded at: https://www.mdpi.com/article/10.3390/cancers15153785/s1, Ingenuity full analysis results of Disease and Function. File name: IPA_output_user_background_030723.xls.
